# Evaluating the efficiency of coarser to finer resolution multispectral satellites in mapping paddy rice fields using GEE implementation

**DOI:** 10.1038/s41598-022-17454-y

**Published:** 2022-08-01

**Authors:** Mirza Waleed, Muhammad Mubeen, Ashfaq Ahmad, Muhammad Habib-ur-Rahman, Asad Amin, Hafiz Umar Farid, Sajjad Hussain, Mazhar Ali, Saeed Ahmad Qaisrani, Wajid Nasim, Hafiz Muhammad Rashad Javeed, Nasir Masood, Tariq Aziz, Fatma Mansour, Ayman EL Sabagh

**Affiliations:** 1 Department of Environmental Sciences, COMSATS University Islamabad, Vehari Campus, Islamabad, 61100 Pakistan; 2 Asian Disaster Preparedness Center (ADPC), Bangkok, Thailand; 3 Institute of Crop Science and Resource Conservation (INRES), Crop Science, University of Bonn, 53115 Bonn, Germany; 4 Queensland Alliance for Agriculture and Food Innovation, Hertely Tackle Building (Bldg 83), Glasshouse Road, St. Lucia, QLD 4072 Australia; 5 Department of Agricultural Engineering, Bahauddin Zakariya University, Multan, Pakistan; 6 Department of Agronomy, Faculty of Agriculture and Environment Sciences, The Islamia University of Bahawalpur (IUB), Punjab, Pakistan; 7 Department of Earth and Environmental Sciences, University of Waterloo, Waterloo, ON N2L 3G1 Canada; 8 Business and Economics Faculty, Department of Economics, Siirt University, Siirt, Turkey; 9 Department of Agronomy, Faculty of Agriculture, Kafrelsheikh University, Kafrelsheikh, Egypt; 10 Department of Field Crops, Faculty of Agriculture, Siirt University, Siirt, Turkey; 11 Department of Agronomy, Muhammad Nawaz Shareef-University of Agriculture, Multan, Pakistan

**Keywords:** Computational biology and bioinformatics, Plant sciences, Environmental sciences

## Abstract

Timely and accurate estimation of rice-growing areas and forecasting of production can provide crucial information for governments, planners, and decision-makers in formulating policies. While there exists studies focusing on paddy rice mapping, only few have compared multi-scale datasets performance in rice classification. Furthermore, rice mapping of large geographical areas with sufficient accuracy for planning purposes has been a challenge in Pakistan, but recent advancements in Google Earth Engine make it possible to analyze spatial and temporal variations within these areas. The study was carried out over southern Punjab (Pakistan)-a region with 380,400 hectares devoted to rice production in year 2020. Previous studies support the individual capabilities of Sentinel-2, Landsat-8, and Moderate Resolution Imaging Spectroradiometer (MODIS) for paddy rice classification. However, to our knowledge, no study has compared the efficiencies of these three datasets in rice crop classification. Thus, this study primarily focuses on comparing these satellites’ data by estimating their potential in rice crop classification using accuracy assessment methods and area estimation. The overall accuracies were found to be 96% for Sentinel-2, 91.7% for Landsat-8, and 82.6% for MODIS. The F1-Scores for derived rice class were 83.8%, 75.5%, and 65.5% for Sentinel-2, Landsat-8, and MODIS, respectively. The rice estimated area corresponded relatively well with the crop statistics report provided by the Department of Agriculture, Punjab, with a mean percentage difference of less than 20% for Sentinel-2 and MODIS and 33% for Landsat-8. The outcomes of this study highlight three points; (a) Rice mapping accuracy improves with increase in spatial resolution, (b) Sentinel-2 efficiently differentiated individual farm level paddy fields while Landsat-8 was not able to do so, and lastly (c) Increase in rice cultivated area was observed using satellite images compared to the government provided statistics.

## Introduction

Rice, grown on 12% of the global cropland area^1^, is a major staple food for half of the world's population. Climate change impacts such as global temperatures and changing precipitation dynamics and land cover changes due to urbanization and industrialization negatively affect paddy rice (*Oryza sativa L.*) production. Rice, an important food crop of Pakistan, is also an export product making a valuable foreign exchange. In Pakistan US$1,376 million were earned by exporting rice alone in 2015–16^2^. However, on the downside, rice is a rigorous water crop when associated with other cereal crops. Therefore, water productivity counts a lot for a developing country like Pakistan, which is already a water-stressed country^3^. Consequently, Small-scale farmers, intensify cropping cycles and increase water use to enhance crop production. Paddy crop, with its high water consumption, can also be linked to global water security issues in the future^4^. At the same time paddy fields are a source of methane (CH_4_), and CH_4_ emissions from paddy rice cultivation have incrased from 7 to 8% worldwide between years 2000 and 2016^5^. Since paddy rice fields provide habitat for free-ranging ducks and wild waterfowls, they contribute to avian influenza virus^1^. Therefore, a regular, accurate and appropriate mapping of paddy crop is crucial for achieving food security, tackling climate change, controlling disease transmission, and improving crop management and production^6^. Remote sensing techniques are extensively used to map rice-growing areas at a frequent temporal resolution and to analyze the effects of external variables (e.g., water shortage and climate change) on rice crop^7–9^. remote sensing provides valuable information about agricultural progress and seasonal crop variation, directly linked to the life cycle of paddy rice^10^. Instead of field investigation, which is time consuming, labor intensive, and financially expensive, remote sensing with standardized approaches can be effectively used to analyze the spatial and temporal variability that may affect the life cycle and rice crop health^11^.

Southern Punjab (Pakistan), a region vulnerable to climate change, has elevated poverty levels^12^, and lacks resources to meet its people's needs^13^. Inadequate crop management practices and and advances in climatic variability are main threats to agricultural production in this province. Therefore the region is expected to face elevated poverty levels in the near future. Rice crop, which is the second major crop of Kharif season in terms of area after cotton, is the second most consumed staple food in southern Punjab after wheat^14^. For effective management of rice production and demand, accurate satellite instruments need to be identified so that effective mapping techniques can be implemented in the region^15^. Government officials, agronomists, and researchers often require accurate and timely crop information for producing statistical reports, policy implementation, and advancement in precision agriculture^15^.

Google Earth Engine (GEE), a cloud based repository of satellite imagery and processing capabilities has gained popularity in recent years due to its ability to handle large datasets, and its power to analyze and visualize big data. This platform enables a user to compute and analyze worldwide satellite imagery with its complete spatial and temporal characteristics in an internet-based cloud platform provided free of cost by Google^16^. GEE even strongly supports studies related to earth and its processes. With its onset in 2010, GEE delivers users all satellite imagery, cloud-based computing, and machine learning algorithms to easily process large datasets^17^. In previous studies^1,6,18,19^, researchers have benefited from GEE platform for paddy rice mapping using phenology-based algorithms along with time series of vegetation indices.

Earlier studies^20–23^ support the individual capability of Sentinel-2, Landsat-8 and MODIS multispectral instruments for rice crop classification. However, to date, no study has compared the efficiency of these multispectral instruments in classifying individual paddy rice fields at farm level. Therefore, this study mainly focuses on using the GEE cloud platform to classify rice crops in southern Punjab region (comprised of Multan, Dera Ghazi Khan, and Bahawalpur divisions). The objective is to compare the freely available and GEE integrated coarser to finer resolution multispectral instruments for rice classification using the RF classifier. In this, classification results will be compared based on their accuracy assessment and area estimation. The results will highlight the comparative efficiency of freely available satellite datasets for mapping paddy crop in the region. Furthermore, the resulting area estimations will play a significant role in precision agriculture, eventually helping the decision-making processes.

## Material and methods

### Study area

**Figure 1:**
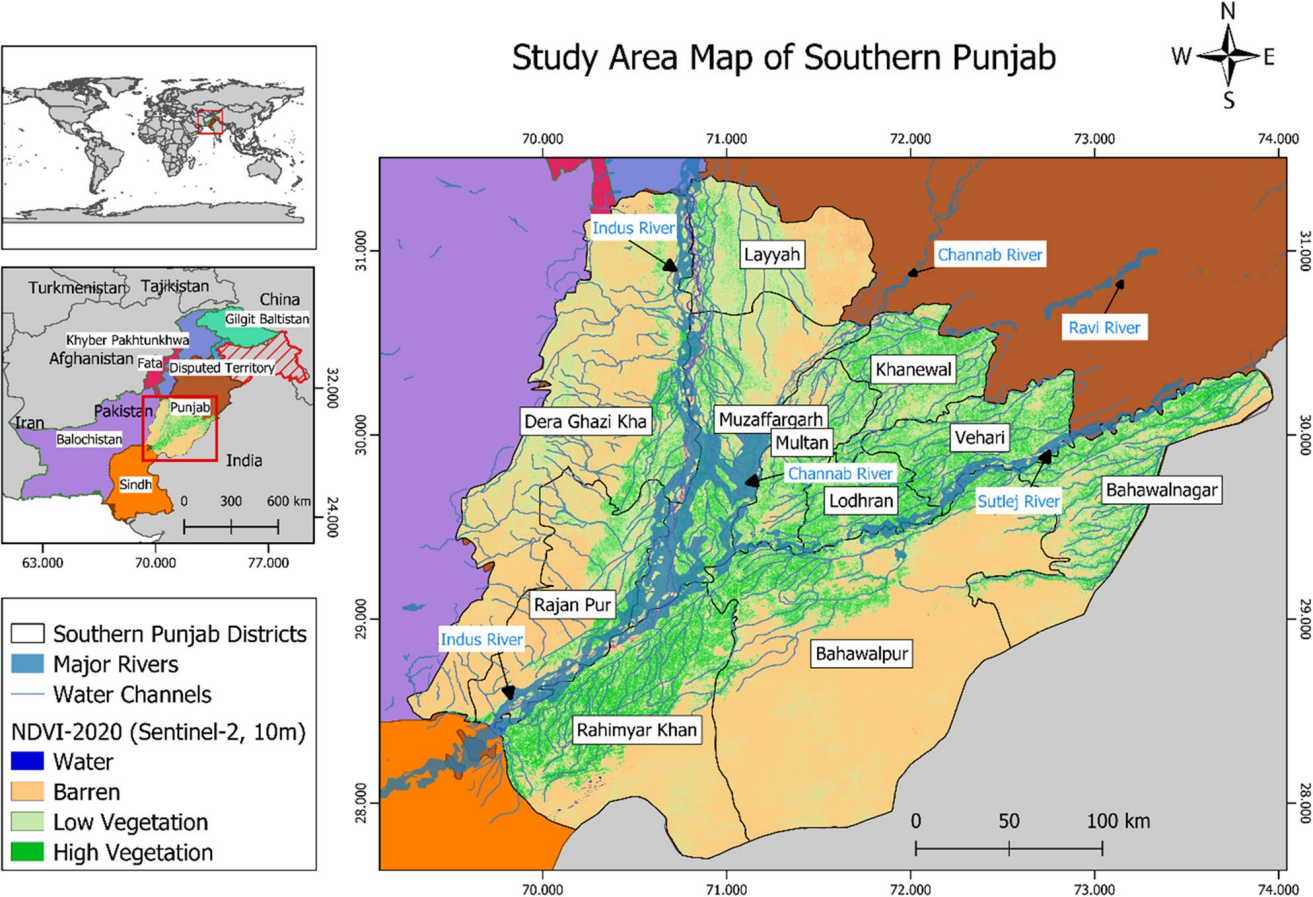
Map of the study area (Southern Punjab). The map is designed in ArcGIS Pro V2.9 software available at ESRI website (https://www.esri.com/en-us/arcgis/products/arcgis-pro/overview). The boundary shapefile used to draw these maps are available at Humdata website (https://data.humdata.org/dataset/cod-ab-pak).

The study area is southern Punjab region (in Pakistan) that lies between latitude 27.30° N to 31.50° N and longitude 69.20° E to 73.55° E (Fig. 1). It has three administrative divisions: Bahawalpur, Multan, and Dera Ghazi Khan. Bahawalpur division has three, while Multan and Dera Ghazi Khan divisions have four districts each^12^. Four major rivers, Chenab, Indus, Sutlej, and Ravi, flow through the study area. Based on visual interpretation, the NDVI derived map shows that the Multan division has higher vegetation than the other two. While Bahawalpur, Dera Ghazi Khan, Rajan Pur, and Layyah districts showed sparse vegetation trends. The southern Punjab region lacks sufficient resources to support its people as the majority of areas are undeveloped with weak infrastructure^13^. The gravity of the situation can be understood by the fact that out of 40 million people in Pakistan living under poverty line, with 10 million of those belong to the Southern Punjab region^12^. In the region, most of the population depends on agriculture for their livelihoods. Few districts, such as Bahawalnagar and Vehari, contain a dense network of water channels, making them suitable for rice production. Additionally, the Bahawalnagar district had high water table than other districts, which makes it fit for rice crop in the Kharif and not suitable for other crops such as cotton^24^. In southern Punjab, there are two main cropping seasons: Kharif (April-June, Summer) and Rabi (October-December, Winter). Rice is the dominant crop of the Kharif season^4^.

**Figure 2:**
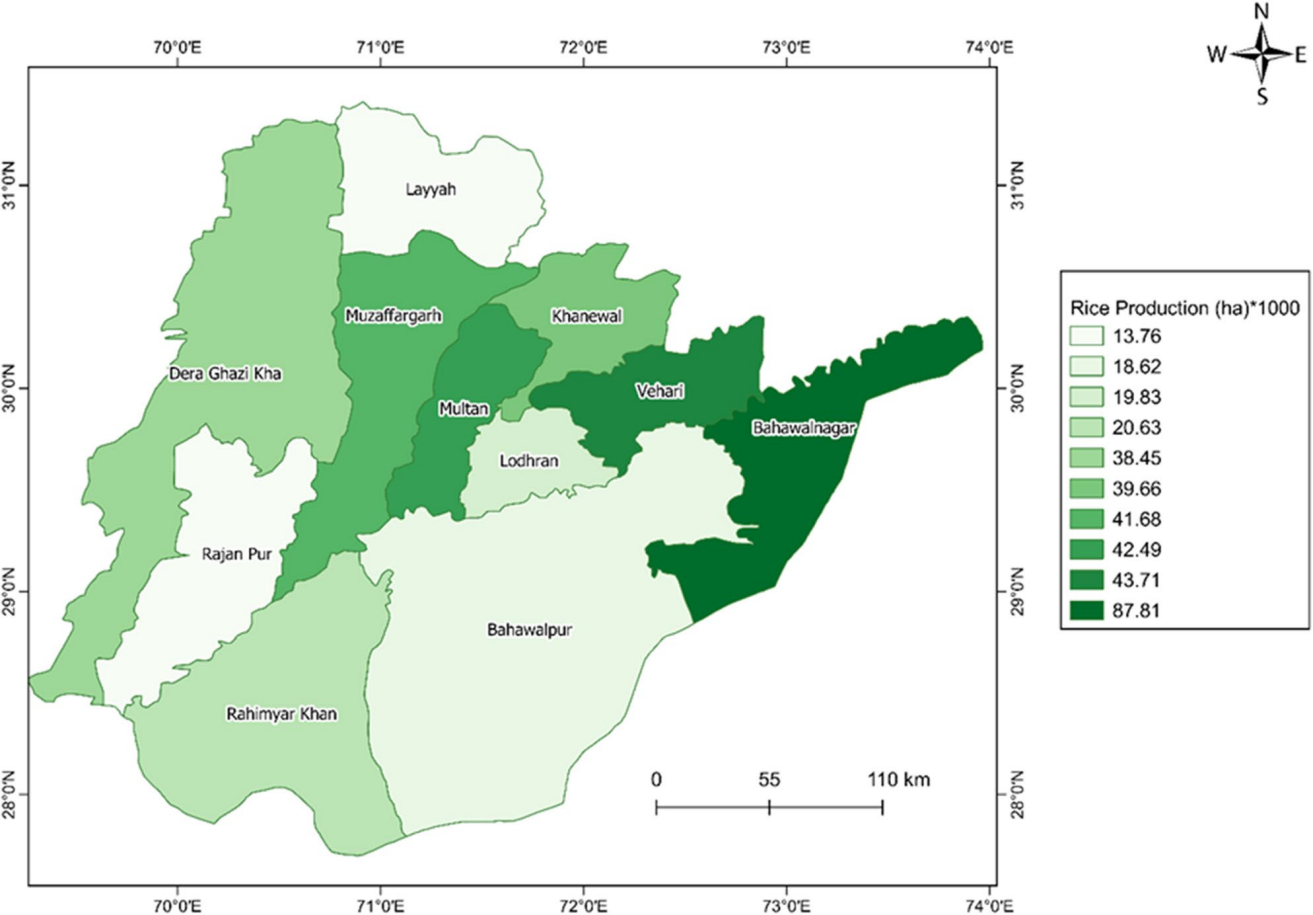
Ground truth data for rice production in southern Punjab for year 2020 (Reported by Crop Reported Service, Government of Punjab). The map is designed in ArcGIS Pro V2.9 software available at ESRI website (https://www.esri.com/en-us/arcgis/products/arcgis-pro/overview). The data used in this map is available at CRS website (http://www.crs.agripunjab.gov.pk).

According to the crop report for the year 2020 provided by the Crop Reporting Service, Department of Punjab (http://www.crs.agripunjab.gov.pk/reports), the total rice area estimation of the Multan, D. G. Khan, and the Bahawalpur division was 380,400 hectares (Fig. 2). The statistical data show that the Bahawalnagar district had the highest area estimate of 87,810 hectares. The lowest area of 13,760 hectares of rice was reported in Rajan Pur and Layyah districts. We use these eastimates for validation of area estimation in our study.

### Data

Satellite imagery in the Near Infrared (NIR), Shortwave-Infrared (SWIR) and microwave region provide valuable information about soil moisture, improving the identification of paddy rice fields^20^. Singha et al*.*^6^ and Wang et al*.*^20^ studies conclude that a combination of normalized difference vegetation index (NDVI) and Sentinel 1 backscatter coefficient (measure of reflective strength of radar body) along with Land Surface Temperature (LST) of day and night time and precipitation data can be effectively use to identify the rice fields during the flooding/transplanting and ripening phases with an overall accuracy greater than 80 percent. Data derived from Sentinel-1 provides valuable information in the microwave region and is used to differentiate paddy rice fields in cloud prone regions because of its capability to penetrate cloud cover. On the other hand, areas without cloud cover issue rely on NIR and SWIR data from multispectral instruments. Similarly, GEE platform is delivering users with coarse to moderate resolution imagery, such as Moderate Resolution Imaging Spectroradiometer (MODIS) at 250 m resolution, Landsat-8 at 30 m resolution, and Sentinel-2 at 10 m resolution. These aforementioned satellite instruments are used in previous studies for paddy rice mapping^1,20–23,25–27^. Sentinel-2 Satellites (2nd series of satellites after Sentinel-1) are part of the Copernicus program initiated by the European Space Agency (ESA)^7^. This satellite mission carries multispectral scanners and consists of two satellites which are Sentinel-2A and Sentinel-2B^28^. In Sentinel-2 satellites, multispectral imaging instruments (MSI) are installed with the ability to record 13 wide-swath bands^29^. Depending upon the band, each satellite provides 10–60 m of spatial resolution with a temporal resolution of 5 days using both satellite constellations. NASA's Landsat-8 mission is a new generation satellite that carries the legacy of Landsat missions. This satellite gets spectral information in visible (V), NIR, SWIR, and Thermal Infrared (TIR) regions^1^. In this satellite mission, two sensors are installed: Operational Land Imager (OLI) and TIR. The OLI obtains spectral information in nine different spectral bands, whereas TIR records thermal information in two bands^30,31^. MODIS mission contains two satellites: Terra and Aqua. These two satellites have nearly the same sensors. The mission monitors the Earth surface every one to two days and obtains spatial data in 36 different spectral bands between 0.405 and 14.385 mm. This mission provides 250 m to 1 km spatial resolution coverage in visible and infrared regions with a 2330 m wide swath^32^.

For preparing paddy rice seasonal spectral profile, Sentinel-2 derived NDVI at 10 m resolution^33^ and Sentinel-1 Backscatter coefficient time-series variation^6^ were accessed and four observations per month (depending upon satellite revisit intervals) were obtained from 10 randomly selected rice fields inside the Southern Punjab boundary. These training points were then digitized inside GEE, and then their monthly average was calculated individually. For average monthly temperature, MODIS 250 m resolution LST dataset was used^34^, and separate day and night LST were calculated. For precipitation, Climate Hazards Group InfraRed Precipitation with Station data (CHIRPS) pentad (5-day) precipitation dataset^35^ was used to calculate average monthly precipitation. The average of LST and precipitation were calculated as average values for the southern Punjab inside GEE.

### Methods

**Figure 3:**
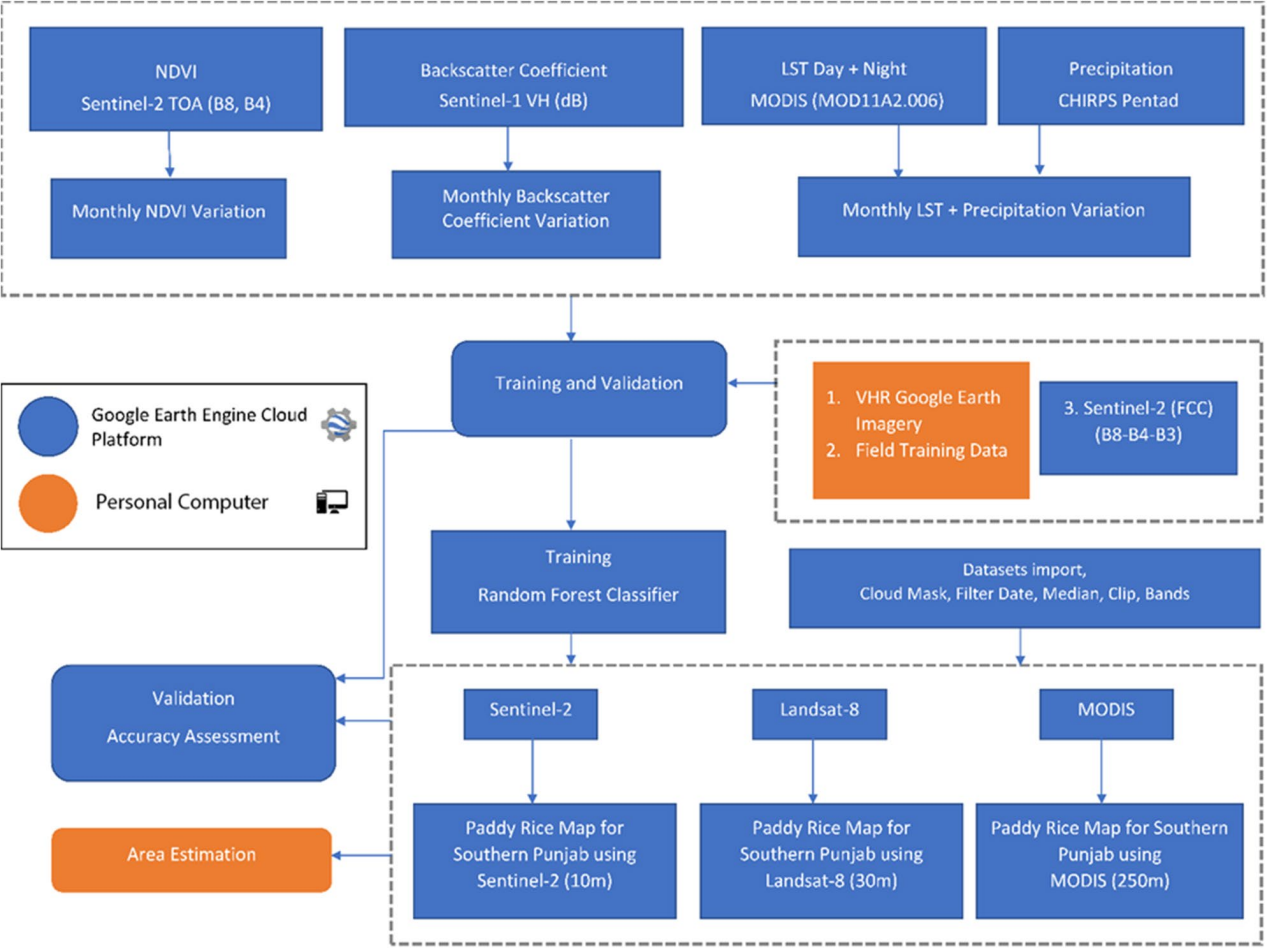
Methodology chart for rice crop classification.

The methodology is divided into three parts. In the first part, rice growth profiles are evaluated using multiple instruments (Sentinel-1, Sentinel-2, Landsat-8, and MODIS), which are crucial to crosscheck training points (non-rice, rice, and water). In second part of methodology, training points (rice, and non-rice) are use to calibrate RF classifier and classify the land cover. After classification, the last part compares derived paddy rice maps using accuracy assessments and differences in the estimated actual areas. The overall methodology performed is shown in Fig. 3. Moreover, all the steps performed in this methodology (i.e., pre-processing and classification) are according to the guidelines and regulations provided by the United States Geological Survey (USGS) (https://www.usgs.gov), and GEE (https://www.explorer.earthengine.google.com/terms).

### Random Forest (RF) based Classification

Random Forest (RF), a machine learning classifier, has widely been used in several studies for paddy rice mapping^36–40^. Because of its high precision, RF (having a usage percentage of 49% globally in satellite image classification) is the most popular classifier, along with Classification and Regression Tree (13%) and Support Vector Machine (11%)^41^. The RF classifier contains a mixture of tree classifiers in which every individual classifier is created using an arbitrary vector tested independently from the user given input, and each of the trees in the classifier gives a vote for the most excellent prevalent class to classify the given input vector^20^. One of the main advantages of an RF classifier is that it needs two types of parameters to be set, while in contrast, the Support Vector Machine need more than two input parameters by the user. Also, the RF classifier can deal with the data without values, which is not conceivable with Support Vector Machine. Another essential advantage of this classifier is that it also offers the comparative importance of different features during the classification process, which is very valuable in selecting features^42^.

Hyperparameter optimization was performed initially which chooses the optimal parameters to be used for algorithm to yield highest accuracy in a given situation. As a result of hyperparameter optimization, max number of trees value was set to 115 which yield maximum accuracy for our study area. Then the trained classifier was used further to classify paddy rice pixels. For classification validation, the training classifier was fed with validation data, and then the resulting trained classifier was used to calculate the error matrix for resultant maps. All these steps, including cloud masking, data filtering, median, clipping, mosaicking, classifier training, classification, and classification validation, were performed individually for each satellite dataset inside GEE.

### Temporal variation of rice using NDVI and backscatter coefficient

Since values of NDVI and Sentinel-1 backscatter coefficient vary with the variation in rice growth stages, they were used inside GEE to track rice growth stages for 2020^6^. Rice crop goes through three distinct growth stages: (1) flooding or transplanting phase; (2) high vegetation period; (3) the harvest period^4,14,43,44^. In the Punjab region, rice growing starts from the mid-May with harvesting from September to November^4,45^. The phenology of rice is shown in Table 1. These development stages are the major reason for variation in NDVI and Sentinel-1 backscatter values.

**Table 1:** Rice crop calendar in southern Punjab, Pakistan.

Flooding or transplanting phase		High vegetation period			Harvesting period	
May	June	July	August	September	October	November

**Figure 4:**
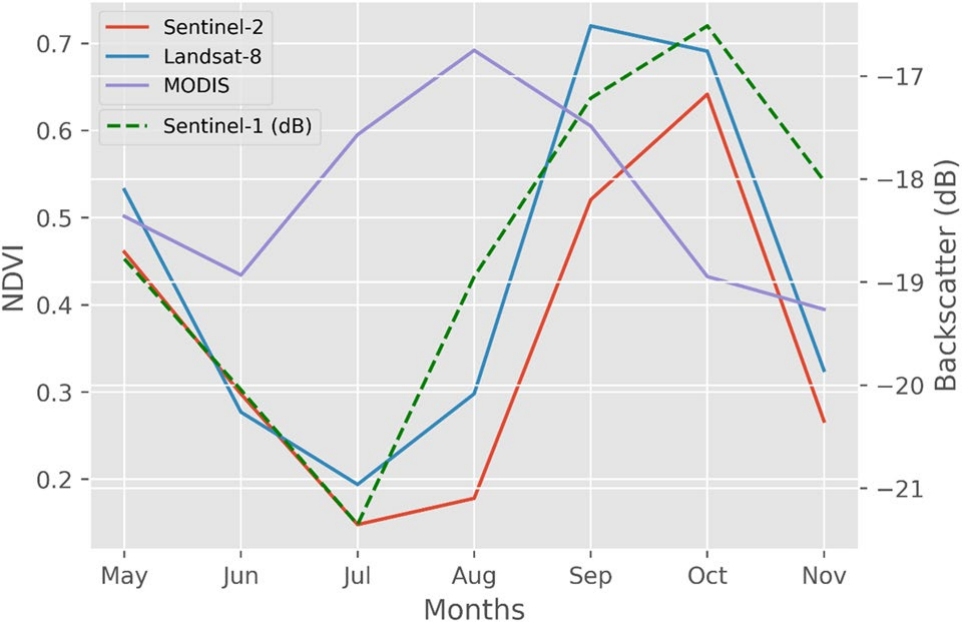
Average NDVI temporal profile for rice using Sentinel-2, Landsat-8 and MODIS derived NDVI data and average Sentinel-1 backscatter coefficient (dB) temporal profile for rice.

To map the temporal variation of rice, we used a literature based methodology^6^. The monthly time series of NDVI was created using Sentinel-2 (10 m), Landsat-8 (30 m), and MODIS (250 m) satellite data (Fig. 4). For comparison, the backscatter coefficient (dB) time series was created using Sentinel-1 in dual-band cross-polarization with vertical transmit, horizontal receive (VH) (Fig. 4). The NDVI variation was observed for rice using three multispectral instruments (Fig. 4). Given the rice crop sowing and transplanting phase, NDVI values of Sentinel-2 and Landsat-8 are low (~ 0.16 to 0.20) in months of June and July. However, the values are comparatively higher (~ 0.51 to 0.55) in September and October, representing higher vegetation. For MODIS, peak values of NDVI were observed in August. From Fig. 4, it was observed that backscatter values were low (~ − 18 to − 21 dB) for the flooding stage of rice due to the presence of water and for the harvest stage due to surface scatter from the open land. In contrast, they were significantly high (~ − 16.51 dB) for the vegetation stage due to volume scattering in the rice plant.

**Figure 5:**
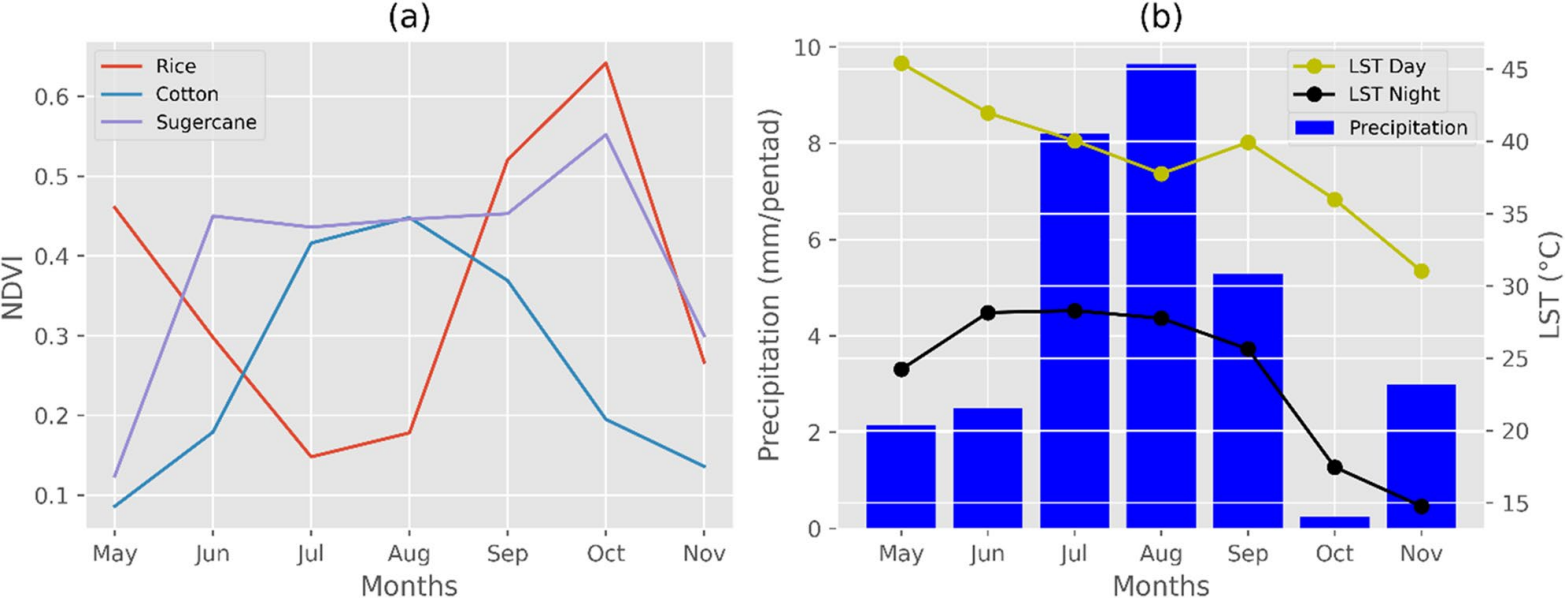
Crops NDVI temporal variation using Sentinel-2 (**a**) and Average Monthly LST (Day and Night) along with CHIRPS precipitation temporal variation (**b**), for 2020 Kharif season in Southern Punjab region.

NDVI variation of various Kharif season crops (Rice, Cotton, and Sugercane) using Sentinel-2 MSI data were compared (Fig. 5a). From their variation, it was observed that NDVI values of crops differ spatially and temporally, therefore, provide great potential in differentiating the crop's growth stages. This phenomenon was also confirmed by previous studies^46–50^. In the last, Fig. 5b shows average day and night LST derived from MODIS satellite along with CHIRPS precipitation data. We used Landsat-8 (100 m thermal data) to derive LST for both day and night. Precipitation time series was prepared using CHIRPS data. CHIRPS is a precipitation dataset which provides 35 years of grided precipitation data with a spatial resolution of 5.5 km^51^. The purpose of LST and precipitation data (Fig. 5b) was solely to verify the climatic condition for the year 2020 which helped us in planning survey and collecting field samples, in appropriate growth cycle of rice crop in souther Punjab. Figure 5b shows that mean precipitation was high during July to September with moderate mean LST. Literature shows that such weather condition is favorable for enhanced rice growth^52^.

### Training samples

Sentinel-1 derived Synthetic Aperture Randar (SAR) data was used to check training points before feeding them to classifier for classification. Previous studies have reported, that the SAR backscatter value is sensitive to water^21^. As paddy rice follows different growth stages, SAR based backscatter can be used to track any pixel either paddy or non paddy. Same principle was applied in this study to differentiate non-rice training points from paddy rice. For specifically non-rice class, each training point was cross checked using Sentinel-1 SAR backscatter data. Only samples which does not follow spectral trend as shown by paddy rice (Fig. 4) were used for representing non-rice training class. To ensure proper coverage and correct selection of the ground truth data for our large study area, we collected the ground truth data (using random sampling approach) through multiple sources: (1) Very High Resolution VHR images from the Google Earth, (2) 167 field rice latitude longitude coordinates point samples randomly collected during the rice field survey in October–November, and (3) Visual interpretation of Sentinel-2 false color composite (FCC) at 10 m spatial resolution.

**Figure 6:**
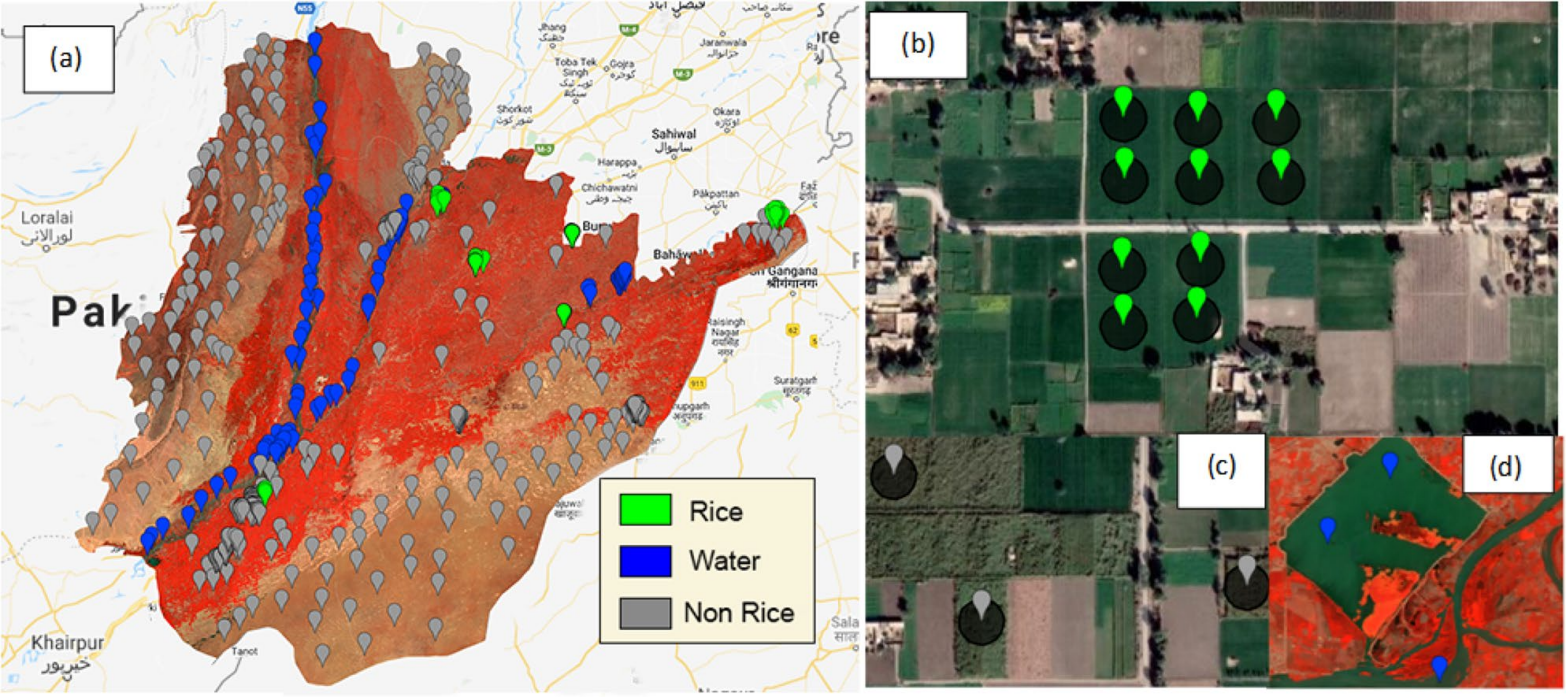
Training samples in the study area, (**a**) all samples, (**b**) ricefield samples, (**c**) non-rice samples, and (**d**) water samples. The map is designed in Google Earth Engine, which is an cloud computing browser based platform (https://earthengine.google.com).

**Figure 7:**
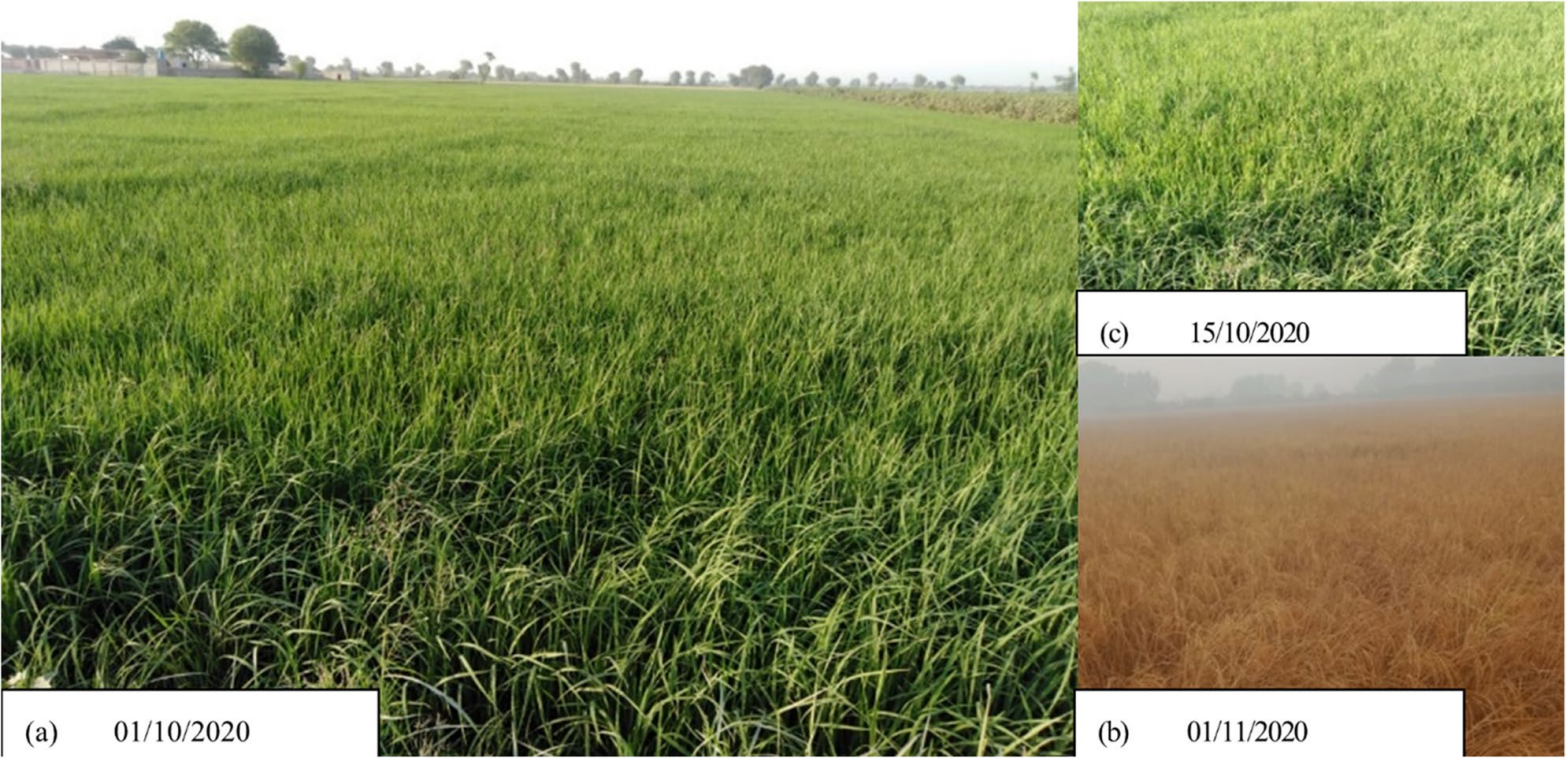
Ricefield photographs, taken during training sample collection at different locations and timeperiod within the study area. The photographs (**a**–**c**) are taken by first author, during the field survey.

Firstly, we used the Sentinel-2 FCC and VHR Google Earth images to digitize points by visual interpretation except for rice (Fig. 6a). Secondly for each class, we made areas of interest AOIs as circle buffers of the points with the radius of 20 m for Sentinel-2 and Landsat-8 (Fig. 6b–d). Thirdly, for rice, we took accurate field coordinates from 6 locations of Southern Punjab and photographs of rice fields at different time intervals are shown in Fig. 7. For collecting rice coordinate samples, we choose 6 random locations in the study area which are shown in Fig. 6a (note the 6 areas where rice points are clustered) and collected samples using random sampling approach. For MODIS, however, it was observed that due to its coarser spatial resolution of 250 m, 20 m buffers was not suitable for training points. So, for MODIS, training points without buffer were used and each pixel contributing to paddy rice class was digitized based on its centroid.

The AOIs without clear land cover information were omitted. The VHR Google Earth imagery and false-color composite combination for Sentinel-2 at 10 m and field photographs are used to clarify the land cover types, i.e., water, barren, forests, non-paddy, and urban areas. The classes, including non-paddy agriculture, forests, grasslands, urban and barren soil, were merged in one class named non-rice. Water class was kept separate due to its distinct spectral characteristic and was merged with non-rice class after training classifier. The reason for separating water class initially from other land-use classes was to restrict water pixels to create confusion during classification^53^. In this study, separating water pixels from paddy rice was crucial as they both share similar spectral characteristics^6^ (only when rice is in flooding growth stage). The field sizes, shapes, and proximities were considered in labeling land cover information of AOIs.

A total of 690 AOIs were collected, including 167 rice samples, 88 water samples, and 435 non-rice samples from different locations of Southern Punjab. The AOIs were distributed randomly, covering the entire study area. The generated AOIs were randomly split (by category) into a 70/30 ratio; 70% were used as training data, while the remaining 30% were used for validation. The paddy rice classification was performed individually for each multispectral satellite dataset utilizing the same training AOIs and the same year (2020).

### Pre-processing

Sentinel-2, Landsat-8, and MODIS datasets (freely available inside the GEE data catalog) were used separately and individually for paddy rice classification. Parameters that were kept the same for a fair comparison among these three datasets were (1) same training, and validation data, (2) same satellite data acquisition dates were used (July–October), and the median was taken to ensure best pixel coverage^6^, (iii) Similar spectral bands (if present) were utilized, (4) The same RF classifier was used in each classification, and in the last, (5) same procedure was applied for accuracy assessment and area estimation.

These datasets were used inside GEE. First, these datasets were used with a cloud mask individually, ensuring minimum cloud coverage in data^54^. Then images between July and October were filtered, and their median was taken. Then the datasets were clipped to the boundaries of the study area. For Sentinel-2 and Landsat-8, Visible band with NIR and SWIR bands are used, because NIR and SWIR bands of Sentinel-2 and Landsat-8 enhance crop classification^55^. However, for MODIS, band-1 SWIR and band-2 NIR images with a spatial resolution of 250 m are used^56^.

## Results

### Rice maps using Sentinel-2, Landsat-8 and MODIS

**Figure 8:**
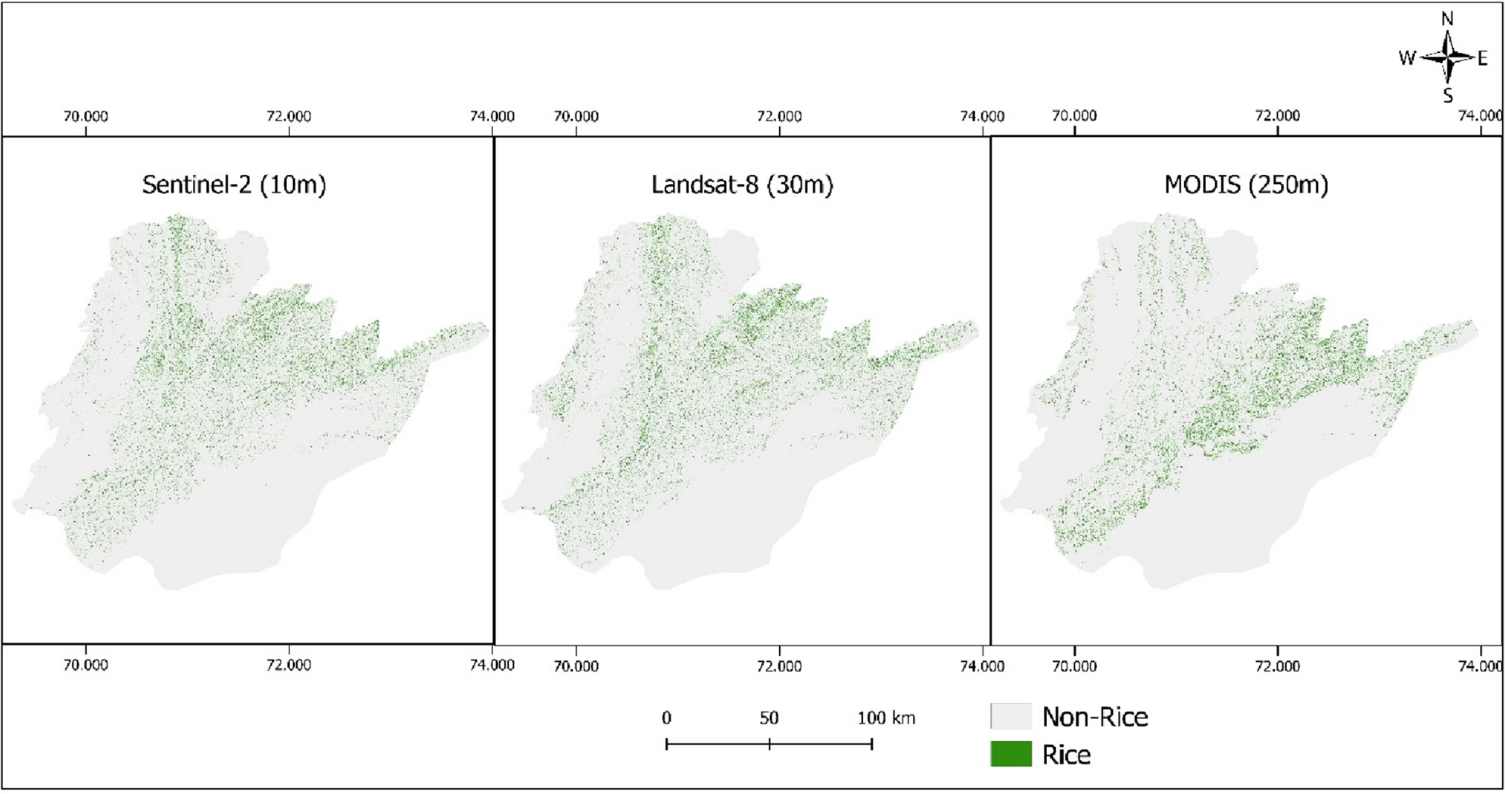
Rice crop map in southern Punjab for the year 2020 using Sentinel-2, Landsat-8 and MODIS datasets.

The rice classified maps for Southern Punjab are generated using Sentinel-2, Landsat-8, and MODIS satellite data (Fig. 8). From their visual comparison, it is observed that MODIS derived maps form clusters instead of classifying individual rice fields, which can be attributed to a coarse-resolution of 250 m. As a result, most pixels of the MODIS map are misclassified because it mixed neighboring crops, and subsequently formed large clusters indicating them rice. Another problem observed in MODIS classified map is denser rice classified pixels clustered in the northeast of southern Punjab, especially in Bahawalnagar, Bahawalpur, and Rahim Yar Khan districts (Fig. 8). This is because of dense vegetation in those areas that can be visually observed in Fig. 1. When compared with Fig. 1, it is confirmed (through visual interpretation) that those areas were actually other crops (Fig. 1) which MODIS has classified totally as rice (Fig. 8).

**Figure 9:**
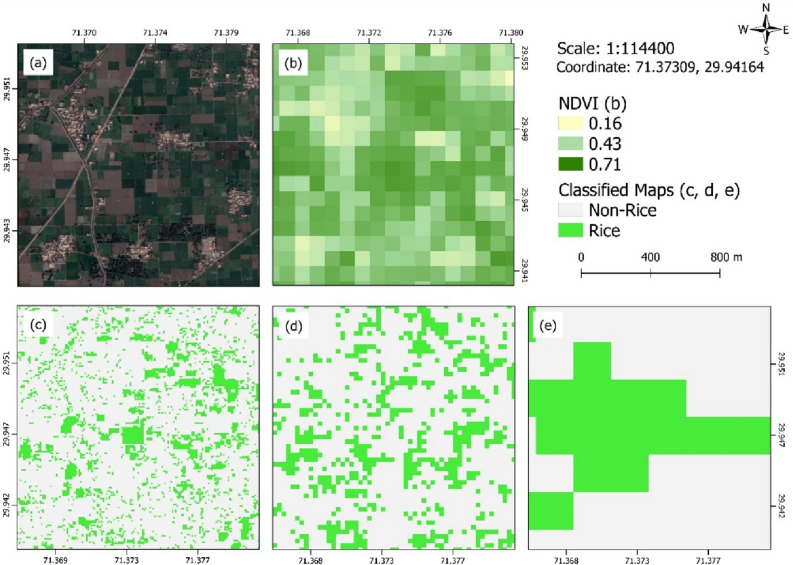
Comparing classified maps at a random location in Southern Punjab using VHR Google Earth imagery (**a**), Sentinel-2 10 m derived NDVI (**b**), rice classified map using Sentinel-2 (**c**), Landsat-8 (**d**) and MODIS (**e**).

Landsat-8, on the other hand, performed better than MODIS. At some points, it was successful in classifying individual rice fields. Paddy crop is mostly grown in small, segmented fields; therefore, spatial resolution plays a great role in identifying individual paddy fields^57^. Landsat-8 misclassified some pixels as rice clusters in Khanewal and Dera Gazi Khan districts, that can be clearly seen in the Fig. 8. The rest of the Landsat-8 data is reliable enough for further use. Sentinel-2 classified individual paddy rice with greatest precision as compared to the other two datasets, and successfully differentiating individual paddy rice fields at a resolution of 10 m. The performance of these datasets in differentiating paddy rice fields is presented in Fig. 9. For visual comparison, VHR Google Earth imagery (Fig. 9a) and Sentinel-2 derived NDVI at 10 m resolution (Fig. 9b) were used as reference images while derived rice classified maps for Sentinel-2 (Fig. 9c), Landsat-8 (Fig. 9d), and MODIS (Fig. 9e) were compared. At a scale of 1:114,400, a random point (with coordinates 71.37309, 29.94164) was selected and the corresponding derived maps (Fig. 9c–e) were compared at this view. It shows that MODIS formed a large cluster and is not able to identify individual paddy fields. On the other hand, Landsat-8 has identified some individual paddy fields, however, it has misclassified the majority of neighboring small pixels as rice. Comparatively, Sentinel-2 has efficiently classified individual rice fields with lowest number of misclassified pixels. Because of its high resolution of 10 m, Sentinel-2 classified individual paddy fields better than both the Landsat-8 and MODIS.

### Technical validation

Through visual inspection (Figs. 8, 9) and accuracy assessment (Table 2), we find that the MODIS-derived map is not reliable for differentiating individual paddy fields and cannot be used in local scale regions decision-making. As for the nearly similar area of Sentinel-2 and MODIS datasets, Sentinel-2 derived map was justified through accuracy assessment and visual interpretation. However, MODIS failed to withstand accuracy assessment and visual interpretation mainly due to cluster formation and huge misclassification.

**Table 2:** Error matrix of paddy rice classification for three satellite data (*UA* user’s accuracy, *PA* producer’s accuracy, *OA* overall accuracy).

Satellites	Class	Error matrix		UA (%)	PA (%)	F1-score (%)	Kappa (%)	OA (%)
		Non-rice	Rice					
Sentinel-2	Non-rice	1712	39	97.7	97.7	97.7	81.2	96.0
	Rice	40	205	84	83.7	83.8		
Landsat-8	Non-rice	1683	26	97.8	98.5	98.1	73.3	91.7
	Rice	38	189	77.9	73.3	75.5		
MODIS	Non-rice	1663	28	93.5	98.3	95.8	61.2	82.6
	Rice	116	137	73	54.1	65.5		

Acuracy assessment is important to check the reliability of land use classification and error matrix is a common method to assess the accuracy^58^. An error matrix is also used to draw other statistical measures of accuracy such as overall accuracy (OA), commission error (i.e., user's accuracy (UA)), omission error (i.e., producer's accuracy (PA)), and the kappa coefficient (K)^59^. The UA evaluated by dividing the number of correctly classified pixels (each cateogory) by the total number of classified pixels in that land use category (the row total)^60^. The UA actually shows the probability that the particular pixel of land-use class of classified map actually represent that land-use class on ground. The equation used to derive UA is given as Eq. 1.

1 $$\mathrm{UA}=\frac{\mathrm{Total\,number\,of\,correct\,classification\,per\,class}}{\mathrm{Row\,total}}$$

The PA is computed by dividing the number of correctly classified pixels in each land-use class by the number of reference pixels in that land use category (the column total)^60^. The PA represents how accurately reference pixels of land-use class are classified and the equation used to derive PA is given as Eq. 2.

2 $$\mathrm{PA}= \frac{\mathrm{Number\,of\,correctly\,classified\,pixels}}{\mathrm{Column\,total}}$$

The overall accuracy (OA) asseses the match or mismatch between classified and ground truth of land use, and is derived by dividing total number of accurately classified pixels by total number of reference pixels^50^. The equation used to derived OA is given as Eq. 3.

3 $$\mathrm{Overall\,accuracy }= \frac{\mathrm{Number\,of\,pixels\,classified\,correctly}}{\mathrm{Number\,of\,reference\,sampling\,pixels}}$$

The Kappa coefficient evaluates the reliability of classified raster which is calculated using error matrix. The coefficient values vary between 0 and 1, where a higher value means a better reliability and vice versa. The Kappa coefficient is calculated using Eq. 4^50^.

4 $$K=\frac{(\mathrm{percent\,overall\,correct\,value }-\mathrm{ percent\,correct\,agreement\,to\,observed\,values}) }{(\mathrm{total\,number\,of\,class }-\mathrm{ percent\,correct\,agreement\,to\,observed\,values})}$$

F1-score or F-measure is a useful statistical approach to find the accuracy of classification using precision and recall^61^. F1-score is a harmonic mean of precision and recall, given in Eq. 5.

5 $$\mathrm{F}1-\mathrm{score}=2\times \frac{\mathrm{Precision }\times \mathrm{ Recall}}{\mathrm{Precision }+\mathrm{Recall}}$$

where precision, also known as the positive predictive value, is calculated by dividing the number of true positive values by the number of all (true and false) positive values (Eq. 6). Recall, also known as sensitivity, is calculated (Eq. 7) by dividing the number of true positive values by total the number of predicted values (true positives plus false negatives)^62^.

6 $$\mathrm{Precision}= \frac{\mathrm{True\,Positives}}{\mathrm{True\,Positives}+\mathrm{False\,Positives}}$$

7 $$\mathrm{Recall}= \frac{\mathrm{True\,Positives}}{\mathrm{True\,Positives}+\mathrm{False\,Negatives}}$$

The classification process is only considered reliable if it meets accuracy checks, because land use land cover (LULC) maps derived from satellite images may contain some errors due to several factors ranging from techniques in classification to satellite based data retrieval methods^63,64^. For paddy rice maps (Fig. 8), a confusion matrix was developed containing kappa coefficient (K), F1-Score, UA, PA, and OA. It shows that OA was more than 90% for Sentinel-2 and Landsat-8 datasets which supports the reliability of derived maps (Table 2). For MODIS, OA was 82% showing least reliability for rice mapping compared to Sentinel-2 and Landsat-8.

### Area estimation

**Figure 10:**
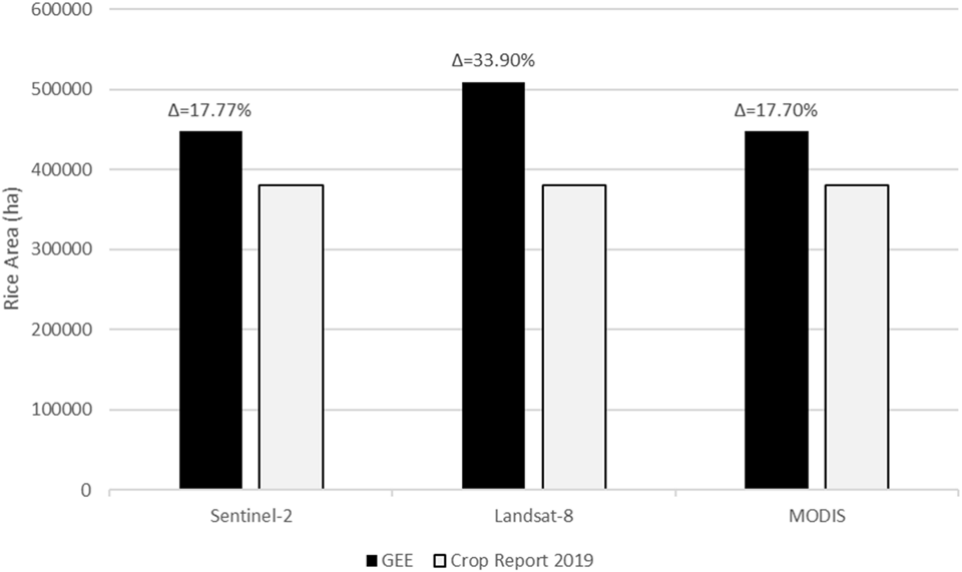
Comparison of rice classified cultivated area from GEE and Crop statistics report (local agrarian data). The percentage of overestimation (Δ) by GEE classification relative to ground truth data is provided for each dataset.

The area of rice crop is calculated from the classified maps using the *“ee.Image.pixelArea()”* function available inside GEE. This function computes and assign area for each pixel, and assign area values to each pixel. Then, we apply *ee.Reducer.sum* which computed total area per class of each classified rice map. For area comparison, crop reported area provided by Crop Reporting Service, Government of Punjab (http://www.crs.agripunjab.gov.pk/reports) was used as ground truth data to calculate mean difference between estimated and reported areas (Fig. 10). Multispectral instruments and ground truth data show a good agreement for estimated rice production. The mean percentage difference was approximately 17% for Sentinel-2 and MODIS while 33% for Landsat-8. Previous studies also reported overestimation^37,65,66^, which is mainly because of misclassified pixels in the rice class that may have contributed to some rise in the classified area.

**Figure 11:**
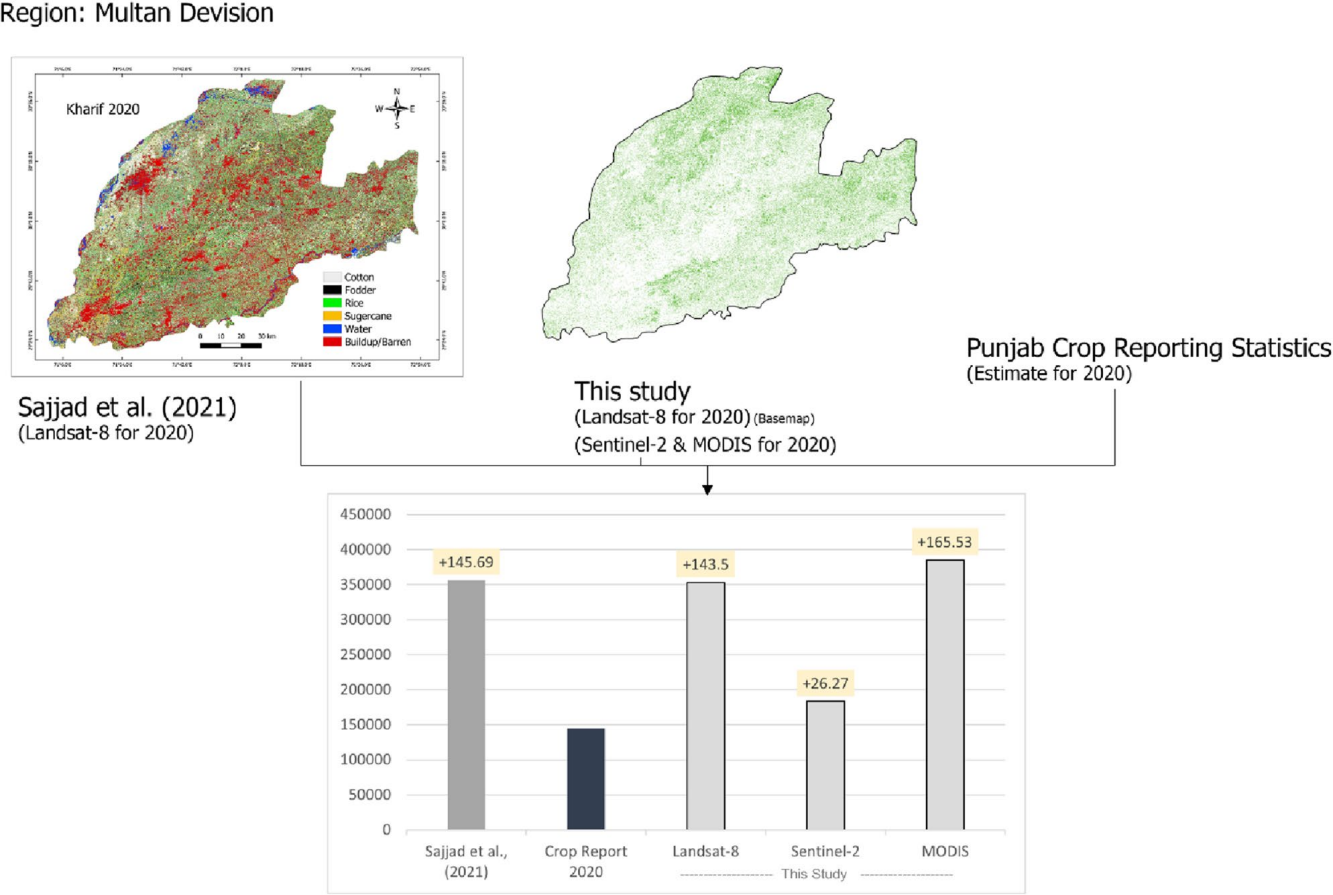
Comparing our rice estimated area using three instruments with previously published study ^70^ and crop statistics report for 2020. Note: in the Figure area is compared for Multan division, and call-outs in bar plots shows the percentage increase or decrease in area compared to crop statistics report. The Figure is designed in Photoshop V23.3 software provided by Adobe (https://www.adobe.com/products/photoshop).

For Landsat-8, the mean percentage difference was more significant (greater than 30%). This overestimation was due to higher misclassified rice pixels as observed previously through visual interpretation of Fig. 8. Surprisingly, the MODIS estimated area was nearly equal to the Sentinel-2 derived area; which reflects why MODIS is used by government officials and many researchers^67–69^ for large-scale LULC area estimation. To further differentiate the performance of each instrument, and to address the issue why MODIS and Sentinel-2 derived paddy rice area was similar, we compared our study results (fosucing on Multan division) with recently published study performed by Sajjad et al*.*^70^ on crop classification in Multan. The Fig. 11 shows the comparison of paddy rice area from our study for Multan division with area published by Sajjad et al*.*^70^ and Punjab crop statistics report for 2020. The Fig. 11 also shows the mean differences between different area sources compared with the base area estimate provided by Punjab crop statistics report for Multan region. From Fig. 11 it was observed that paddy rice area from Sajjad et al*.*^70^ study using Landsat-8 also showed overestimation (+ 145.7%) whereas our derived area using Landsat-8 also shows similar overestimation (+ 143.5%). Furthermore the Fig. 11 also highlighted a major difference in area estimation of Sentinel-2 (+ 26.3%) and MODIS (+ 165.5%) compared with Punjab crop statistics report.

## Discussion

Rice mapping is essential for bringing water-use efficiency, achieving regional food security, controlling disease transmission, and countering global warming. As our study area is a semi-arid region with lower precipitation (Fig. 5b), free available and GEE integerated MSI are suitable to map paddy rice crop due to lower cloud cover in the cropping months^27,71,72^. Despite their frequent use in mapping paddy fields, no study has compared the efficiency of Sentinel-2, Landsat-8 and MODIS datasets in classifying individual paddy rice fields. Our study compares these datasets by estimating their paddy crop classification potential, using accuracy assessment methods, and the mean difference between estimated area and area based on ground truth data.

The NDVI values derived from Sentinel-2 at 10 m spatial resolution (Fig. 5a) were used to track changes in crop growth stages. The NDVI of any crop differs spatially and temporally and thus can be used as a valuable tool to study crop growth changes over space and time^48,49,71,73,74^. The temporal variation shows that rice sowing and transplanting stages start from May until June in southern Punjab region. Lower NDVI values (Fig. 5a) in July and August reflect significant NIR adsorption on the water surfaces which in this case are paddy rice fields flooded with water and with minimum green area. Similarly, Usman et al*.*^50^ stated that the individual crops starting time and crop-cycle length can be visualized easily from NDVI trends. According to them, low positive values of NDVI (0.1 or less) represent barren areas of rock, sand, or snow, while 0.1–0.2 represent soils. Most vegetation has moderate NDVI values (~ 0.2 to 0.5), while dense forests show high NDVI values (~ 0.6 to 0.9). The class 'rice' exhibits a unique trend compared to other crops (Cotton, and Sugarcane) in the Kharif season (Fig. 5a). For rice crop, the initial NDVI variation is a bit slow and lengthy because of rice nursery growth in June. However, the latter part of NDVI variation reaches its maximum due to rapid rice crop growth in September and October. Figure 7b shows variation in temperature and precipitation patterns during the Kharif season. It was observed that from July to September, mean precipitation values were high along with moderate LST (Day and Night), which supported rice cultivation^14,52^.

For rice crop, Sentinel-2 and Landsat-8 data showed similar NDVI trends (Fig. 4). Due to their high spatial and temporal resolution, these trends were successful in differentiating various rice growth stages. Conversely, the MODIS curve showed a peak response in August, mainly due to its coarse resolution (of 250 m). Although MODIS has a higher revisit time (1 day) than Sentinel-2 (5 days) and Landsat-8 (16 days), it cannot be used to study the growth cycle of individual rice fields due to its coarser resolution. Likewise Chen et al*.*^75^ concluded that the MODIS imagery with 250 m resolution could not provide accurate vegetation information on corn growth. The variation in Sentinel-1 backscatter coefficient for paddy rice (Fig. 4) showed that the coefficient is very sensitive to water in paddy rice fields. The backscatter values were low in June and July due to the presence of water in the fields, and were high in September and October due to high vegetation. Singha et al*.*^6^ noted a similar trend in the the backscatter values. The Sentinel-1 VH backscatter coefficient can be used as an indicator for tracking paddy rice growth as the backscatter values change with the varying conditions of paddy rice stages. Unlike other crops, rice spends a substantial time submerged in water. Thus, it can be easily differentiated from other crops. Also, it has distinct growth stages, which can be observed using the Sentinel-1 VH backscatter coefficient. Based on the temporal variation of NDVI and Sentinel-1 backscatter coefficient, an accurate rice growth profile was generated for the Southern Punjab region for the year 2020, which was used as a baseline in rice fields sampling. The coordinates used for rice training and validation by global positioning system (GPS) were crosschecked using NDVI and backscatter temporal variation. This step was performed inside GEE to ensure that only rice fields coordinates having the same trend as observed in Fig. 5 were taken for further classification and accuracy assessment. Previous studies^6,20,76–78^ support the RF classifier's capability in identifying paddy fields. Therefore, this study utilized RF classifier for the classification of paddy rice.

Results showed that Sentinel-2 performed best in classifying individual rice fields (Figs. 8, 9). MODIS failed to accurately classify rice fields, and it formed clusters with neighboring fields. A visual interpretation was performed for comparing the efficiency of each dataset in classifying individual rice fields. It was observed that Landsat-8 efficiently differentiated paddy rice fields; however, it misclassified rice pixels at some points with other crops having similar vegetation characteristics. Overall, Sentinel-2 performed best in accuracy assessment than both Landsat-8 and MODIS (Table 2).

Further, we compare estimated and reported areas for rice production, which shows an overestimation of ~ 18% for both Sentinel-2 and MODIS, and ~ 34% overestimation for Landsat-8 (Fig. 10). Similar overestimation was reported by other researchers^37,65,66^. For example, Mananze et al*.*^37^ used GEE to map shifting agriculture dynamics in Mozambique and found 15.5% of mean difference between the estimated and ground truth areas for agricultural land use. In another study to map cropping intensity of small-scale farms in India, Jain et al*.*^79^ reported a large discrepancy (with percentage difference of 150.8%) between cropland area estimated through remote sensing (Landsat data) and the area obtained from agricultural census. Likewise, Dheeravath et al*.*^80^ found that irrigation area derived using MODIS (500 m) data was greater than the ground truth data (with the mean percentage difference of 17.2%) in most Indian States.

To compare the overestimation in paddy rice area, we compared our results with a previous study performed by Sajjad et al*.*^70^ focusing on Multan division (Fig. 11). The Fig. 11 shows area estimated as well as mean percentage difference of area from each source compared with crop statistics report. It was observed that there exist a similarity in overestimation of Landsat-8 derived area reported by Sajjad et al. (2021) (145.7%) and our Landsat-8 derived paddy rice area for Multan division (143.5%). The Punjab crop statistics report provided area was taken as base area for comparison. When compared, Sentinel-2 with mean percentage overestimation of 26.3% stands above Landsat-8 and MODIS with overestimation 143.5% and 165.5% respectively. To conclude, MODIS performance was least, as this instrument overestimated the area for Multan division with an overestimation of 165.5%, far above the percentage difference of Landsat-8. This reflects why for crop area estimation MODIS should be avoided and preference should be towards Sentinel-2.

The multispectral imagery-based paddy rice mapping implemented in GEE includes several benefits. First, there is reduction in data acquiring and pre-processing time^40^. Otherwise, it might have taken longer to download and pre-process the datasets for a large study area like ours (i.e., southern Punjab). Second, there is a significant decrease in computational time^81^. The MSI-based RF algorithm inside the GEE performed paddy rice fields classification in a very short time. Thus, high-performance computing resources like GEE facilitate quick and rapid mapping of paddy rice planting areas at a large scale^1,6^. Future studies of paddy rice mapping under similar climatic conditions can utilize the most efficient dataset found in this study: Sentinel-2. In addition, further research may focus on rice mapping for the entire country using high-resolution Sentinel-2 imagery with a similar GEE-based methodology.

## Conclusions

In this study, we compared three, free and open, satellite datasets (Sentinel-2 (10 m), Landsat-8 (30 m), and MODIS (250 m)) to evaluate their efficiency/effectivity for mapping of rice crop in southern Punjab, Pakistan. The results show that rice classification improves with increase in spatial resolution. Among the three, the coarsest resolution dataset, MODIS, do not precisely classify individual rice fields. However, the other two high-resolution datasets (i.e., Sentinel-2 and Landsat-8) can accurately track crop growth stages for making its growth profiles and studying temporal trends. From the visual interpretation, accuracy assessment, and mean difference in area estimation, we observe that MODIS was not able to classify individual rice fields correctly in this case. However, Landsat-8 classified the paddy rice fields accurately with moderate misclassification. In comparison to MODIS and Landsat-8, Sentinel-2 performed best in differentiating individual rice fields with lower misclassification and showed a lesser mean difference in area estimation. Due to limited resources and training data, this study focused only on the RF classifier to classify southern Punjab divisions. JavaScript API is used inside GEE, which facilitates future studies to implement the same methodology in GEE with ease. Future studies may focus on (1) rice mapping of the whole of Pakistan, which could be easily implemented inside GEE using the same methodology and code, (2) comparison of different machine learning classifiers (like Random Forest RF, Support Vector Machine, and Artificial Neural Network to classify paddy rice efficiently, and (3) comparison of Synthetic Aperture Radar and Multispectral Instruments to map paddy rice efficiently.

## Data Availability

All the datasets used in this study are open access, and references are provided at first mention. Paddy rice classification images for Southern Punjab are also available, and can be provided upon reasonable request to corresponding authors. No direct plant material is used in this study. Only open access multispectral satellite images are used, that are freely available inside GEE data catalogue (https://www.developers.google.com/earth-engine/datasets).
